# Predictors of medication nonadherence among hypertensive clients in a Ghanaian population: Application of the Hill‐Bone and Perceived Barriers to Treatment Compliance Scale

**DOI:** 10.1002/hsr2.584

**Published:** 2022-04-13

**Authors:** Eric Woode, Eric Boakye‐Gyasi, Yaa Obirikorang, Evans A. Adu, Christian Obirikorang, Emmanuel Acheampong, Enoch Odame‐Anto

**Affiliations:** ^1^ Department of Pharmacology and Toxicology, School Of Pharmacy University of Health and Allied Sciences Ho Ghana; ^2^ Department of Pharmacology, Faculty of Pharmacy and Pharmaceutical Sciences, College of Health Sciences Kwame Nkrumah University of Science and Technology Kumasi Ghana; ^3^ Department of Nursing, Faculty of Health Sciences Garden City University College Kumasi Ghana; ^4^ Department of Molecular Medicine, School of Medicine and Dentistry Kwame Nkrumah University of Science and Technology Kumasi Ghana; ^5^ Department of Medical Diagnostics, Faculty of Allied Health Science, College of Health Sciences Kwame Nkrumah University of Science and Technology Kumasi West Africa Ghana; ^6^ School of Medical and Health Science, Centre for Precision Health Edith Cowan University Perth Australia

**Keywords:** antihypertensives, Hill‐Bone, hypertension, nonadherence, perceived barriers

## Abstract

**Background and Aim:**

Nonadherence to antihypertensive medication impairs optimal blood pressure and is influenced by multiple interrelating factors. Knowing the complexity of medication nonadherence and its associated factors is essential for intervention strategies. This study evaluated the predictors of medication nonadherence among hypertensive clients in a Ghanaian population.

**Methods:**

This was a hospital‐based cross‐sectional study conducted at the Hypertensive Clinic of the Kwame Nkrumah University of Science and Technology (KNUST) Hospital, Kumasi, Ghana. A self‐designed questionnaire, the Hill‐Bone Compliance to High Blood Pressure Therapy and Perceived Barriers to Treatment Compliance Scales, were used for data collection from 246 hypertensives. Data were analyzed using Statistical Package for Social Sciences, version 25.

**Results:**

Medication nonadherence was observed among 8.5% of the study participants. In a multivariate regression model perceived noneffectiveness of medication (odds ratio [OR] = 1.76, 95% confidence interval [CI]: 1.34–2.31, *p* < 0.001) and barriers to alcohol and smoking cessation (OR = 2.83, 95% CI: 1.31–6.13, *p* = 0.008) were associated increased odds of antihypertensive medication nonadherence. Also, patients who do not know their total prescription (OR = 8.81, 95% CI: 2.28–34.0, *p* = 0.002) were more likely to be nonadherent to their antihypertensive medications. Moreover, clients who associate signs/symptoms of palpitations (OR = 5.82, 95% CI: 1.31–25.80, *p* = 0.021), poor sleep (OR = 3.92, 95% CI: 1.09–14.12, *p* = 0.036) and decreased sexual drive (OR = 4.74, 95% CI: 0.96–23.28, *p* = 0.055), were more likely to be nonadherent to antihypertensive medication.

**Conclusion:**

In conclusion, we observed a lower nonadherence rate among hypertensive clients in a Ghanaian population with correlates being medication‐related factors. Most importantly, perceived noneffectiveness of medication, barriers to smoking and alcohol cessation, palpitations, poor sleep, and decreased sexual drive significantly predicted lower adherence and could serve as indicators for high risk of nonadherence to antihypertensive medications.

## 1 INTRODUCTION

Hypertension (HPT) represents global crises and a single risk factor for cardiovascular diseases. The World Health Organization (WHO) 2019 fact‐sheet indicated that an estimated 1.13 billion people worldwide have HPT, with two‐thirds living in low‐ and middle‐income countries (LMIC).^1^ This figure represents a global increase burden of HPT. Africa shares about 46% of all HPT mortality in LMIC.^2^ In Ghana, the prevalence rates of HPT range from 19.2%–32.8% in the rural population to 25.5%–48.0% in urban centers. HPT was the highest represented disease in the Ghana Health Service 2014 Annual report, between 2011 and 2014. The Ashanti region recorded the second‐highest number of new outpatient hypertensive cases (140,947) diagnosed simultaneously.^2^ More recently, less than half of hypertensive clients in Ghana are aware of their hypertensive status.^3^

With the constantly alarming figures of HPT in general and especially in Ghana, common behavioral changes represent a low‐cost alternative for prevention. Besides, among those living with HPT, diagnosis, initiation, and retention to care and adherence to both pharmacological and nonpharmacological therapy are essential for reducing consequent cardiovascular complications.^4^ Notwithstanding the existence of vast evidence on the advantages of HPT treatment, control of blood pressure (BP) has been disappointing. Various studies have reported that BP control among hypertensive clients in Ghana is mainly poor due to noncompliance to therapy.^5^ Previous studies have established the high prevalence of uncontrolled HPT in Ghana,^2^, ^6^, ^7^ however, there is limited data on correlates that influence HPT control in the Ghanaian hypertensive population.^3^, ^5^ Thus, assessing medication adherence and its correlates is vital to ascertain and monitor treatment and its outcomes to reduce HPT‐related mortality.

It is essential to admit that addressing medication adherence is essential and disreputably difficult in practice. Sometimes, clients are unable to give accurate reports of their adherence to medication. It has been shown that less than 50% of hypertensive patients that are initiated to care, take their medications as prescribed.^3^, ^8^ To‐wit interviewing clients using questinnaires represents a powerful tool for ascertaining clients' concepts and behavior regarding medication adherence. These tools represent an easy‐to‐use, low‐cost subjective method to survey clients' medication‐taking behavior and possible factors that influence their decision. They are designed to standardize and minimize the limitations of other self‐reported adherence measurement methods to a specific medication regime.^9^ Thus, providing basic information that helps optimize treatment and form a therapeutic alliance in which clients' doubts and difficulties with therapy can be detected and addressed. This study used Hill‐Bone Therapy Compliance and Perceived Barriers to Treatment Compliance Scales to evaluate medication nonadherence and its correlates among hypertensive clients in a Ghanaian population.

## 2 MATERIALS AND METHODOLOGY

### 2.1 Study design/site

The study used a descriptive cross‐sectional study design. It was conducted at the Kwame Nkrumah University of Science and Technology (KNUST) Hospital, Kumasi, Ghana. It is a University Hospital located on the premises of KNUST, in the Oforikrom constituency and municipality. In line with the determination of the hospital to provide effective and accessible healthcare services to its clients, the KNUST Hospital established the HPT Clinic, which commenced its activities on May 30, 2008. The main aim of the clinic is for the efficient management of HPT to improve their quality of life and prevent or reduce complications in these clients. The clinic has about 600 registered hypertensives as clients.

### 2.2 Eligibility criteria

Participants who had been diagnosed with HPT and are on treatment for at least 6 months attending the hypertensive clinic of KNUST Hospital and gave written consent were recruited into the study. Clients who have been on treatment for less than 6 months were excluded from the study. Also, clients who were transferred in or not registered at the KNUST hospital for regular appointment schedules were excluded from the study. Clients who had confirmed comorbidities such as diabetes mellitus, heart diseases, renal diseases were also excluded.

### 2.3 Sample size justification

The sample size for this study was calculated using the formula described by Charan and Biswas.^10^ Using 58.6% as the rate of noncompliance to antihypertensive treatment from our previous study,^3^ with a standard normal variate of 1.96 (at 5% type‐1 error), 0.05 precision and 65% response rate, the least estimated sample size for the study was 242 from a total estimated population of 600 clients over 6 months period. Accordingly, a sample size of 246 was finally used.

### 2.4 Data collection

Data were collected from September 2018 to February 2019. Participants were recruited using the convenient sampling method. Depending on the total number of clients per clinic, a range of 15–22 clients were conveniently sampled weekly for the entire 6 months. Hypertensive clients who did not consent or were seriously ill (too sick to be interviewed) were excluded from the study. For all included participants, a questionnaire interview was conducted using a structured close‐ended questionnaire to collect data on demography, therapy adherence, perceived barriers, and symptoms/side effects of medication. The entire questionnaires were available in the English version but were interviewed carefully with the proper translation of the official local language of the study population. The participants' responses were translated back to English in the correct meaning as was interpreted.

### 2.5 Description and validation of questionnaires

#### 2.5.1 Sociodemographic questionnaire

The questionnaire consisted of items that assessed the participants' details and medication history. Besides, the questionnaire contained sections that evaluated medication's most frequently perceived side effects from the past 3 months before data collection.

#### 2.5.2 Hill‐Bone Compliance to High Blood Pressure Therapy Scale

This questionnaire was adapted from a previous study by Kim et al.^11^ that assessed antihypertensive therapy adherence in predominantly black populations. It is a 14‐items scale used to assess patients' behaviors for three important behavioral domains of high BP treatment: (1) reduced‐sodium intake, (2) appointment keeping, and (3) medication taking. In this study, the only influential factor that could be interpreted in acceptability, reliability, and validity analysis was the medication‐taking subscale (Table S1). Thus, we simplified the 14‐items Hill‐Bone Scale to an 8‐items scale for the assessment of medication intake behavior. This eight‐questions item is similar to the short‐form scale used by previous studies.^11^, ^12^ The internal consistency and reliability of the scale were shown by a Cronbach's *α* (0.763) and mean inter‐item correlation (0.243) (Table S1). The total score ranged from 19 to 32 (mean = 28.9797) with higher scores indicating good adherence. A value below or equal to the fifth percentile which is equivalent to ≤80% (mean − 2SD) of the total score was used to define medication nonadherence.

#### 2.5.3 Perceived Barriers to Therapy Compliance Scale

The questionnaire consisted of nine items that assessed perceived barriers to medication adherence. The questionnaire was designed to assess four domains of medication nonadherence; perceived benefits of medication (three items), barriers to medicine accessibility (two items), barriers to lifestyle and dietary practices (two items) as well as barriers to alcohol and smoking cessation (two items). The reliability of the scale ranged from 0.621 to 0.800 (Table S2).

### 2.6 Ethical consideration and informed consent

The Committee on Human Research approved the study, Publications and Ethics (CHRPE), School of Medical Sciences, Kwame Nkrumah University of Science & Technology (KNUST), Kumasi (CHRPE/AP/215/17) and the research committee of KNUST Hospital, Kumasi. Informed consent was obtained from all participants and confidentiality was assured.

### 2.7 Data analysis

Data analyses were performed using SPSS v. 25. Descriptive statistics in percentages and cross‐tabulation were used to evaluate demographic and adherence status. Inferential statistics to examine the relationships between adherence and perceived barriers to adherence were computed using the Spearman rank correlation and logistic regression analysis. Also, univariate and multivariate analysis was used to examine factors associated with medication nonadherence. All factors associated with nonadherence at a *p* < 0.1 were included in a prediction model, and the results were presented. *p* < 0.05 were considered statistically significant.

## 3 RESULTS

A descriptive summary of sociodemographic and medication history is shown in Table 1. Participants aged 50–59 years and 60–69 years constituted 37% and 39% of the study sample. Also, female participants mainly were represented (69.5%). Also, most of the respondents have been on treatment for either 8–10 years (32.5%) or above 10 years (37.8%).

**Table 1:** Sociodemographic characteristics of the study participants

Variable	Category	Frequency (*N* = 246)	Percentage (%)
Age (years)	<50	42	17.1
	50–59	91	37.0
	60–69	96	39.0
	70–79	17	6.9
Gender	Female	171	69.5
	Male	75	30.5
Marital Status	Single	26	10.6
	Married	188	76.4
	Widowed	32	13.0
Level of education	No formal education	41	16.7
	Basic School	112	45.5
	High school	36	14.6
	Tertiary	57	23.2
Occupation	Government employee	47	19.1
	Retired	43	17.5
	Self‐employed	131	53.3
	Unemployed	25	10.2
Religion	Christian	219	89.0
	Muslim	27	11.0
Duration on treatment (years)	<1	14	5.7
	1–3	21	8.5
	4–7	38	15.4
	8–10	80	32.5
	>10	93	37.8

Table 2 summarizes the medication history of the study participants. More than half of the respondents did not know the names of their current medications. Most of the respondents were on dual therapy (36.2%), followed by three‐drug treatment (27.6%). A majority (69.9%) had ever forgotten to take their BP medicine. Frequently perceived signs/symptoms of antihypertensive medications were headaches (26.4%), followed by muscle pain (18.3%), tiredness (11.0%), decreased sexual desire or ability (9.8%), poor sleeping (8.5%), frequent urination (7.7%), palpitations (6.5%), and swollen feet (4.9%).

**Table 2:** Medication characteristics of the study participants

Variable	Category	Frequency (*N* = 246)	Percentage (%)
Knowledge of the name of current medication use	No	133	54.1
	Yes	113	45.9
Knowledge of total prescribed medication	Don't know	47	19.1
	Yes (<3 medicines)	89	36.2
	Yes (≥3 medicines)	110	44.7
Prescription pattern	Monotherapy	10	4.1
	Fixed‐dose combination	34	13.8
	Dual therapy	89	36.2
	Three‐drug therapy	68	27.6
	Four‐drug therapy	31	12.6
	Five‐drug therapy	14	5.7
Appointment periods and blood pressure (BP) check‐ups	<Every 3 months	22	8.9
	Every 3 months	130	52.8
	Every 4 months or beyond	35	14.2
Ever forgotten to take BP medication within the past 3 months	No	74	30.1
	Yes	172	69.9
Perceived signs/symptoms of medication	No	127	51.6
	Yes	119	48.4
Frequently perceived sign/symptom of medication over the last 3 months	Tiredness	27	11.0
	Palpitations	16	6.5
	Swollen feet	12	4.9
	Muscle pain	45	18.3
	Headaches	65	26.4
	Poor sleeping	21	8.5
	Frequent urination	19	7.7
	Low libido	24	9.8

**Figure 1:**
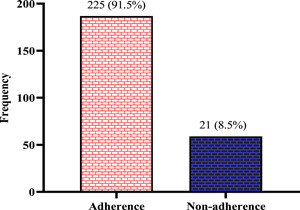
Distribution of medication adherence among the study participants

The response frequencies for the 8‐items Hill‐Bone Medication Compliance subscale and Perception of Barriers to High Blood Pressure (BP) Therapy Compliance Scale are listed in Table 3. Most study participants indicated that they either forget to take their medications (64.2%) or decide not to take their medication (83.3%). The majority of the respondents opted for answers “none of the time” in all domains of the Hill‐Bone subscale. The option “not at all” recorded the highest score on all the fields of the perceived barriers on the compliance scale (Figure 1).

**Table 3:** Responses for each item of the Hill‐Bone Compliance and Perception of Barriers to HBP Therapy Compliance Scales

**Hill‐Bone Medication Compliance subscale (*N* = 246)**				
**Items**	**All of the time**	**Most of the time**	**Some of the time**	**None of the time**
How often do you forget to take your HBP medicine?	0	6.5	64.2	29.3
How often do you decide not to take your HBP medicine?	0	6.9	83.3	9.8
How often do you forget to get prescriptions filled?	1.6	2.0	27.6	68.7
How often do you run out of HBP pills?	0.4	1.2	30.9	67.5
How often do you miss taking your HBP pills when you feel better?	0.8	5.3	25.2	68.7
How often do you miss taking your HBP pills when you feel sick?	0	0	4.9	95.1
How often do you take someone else's HBP pills?	0	0	0.8	99.2
How often do you miss taking your HBP pills when you are careless?	0	0.4	11.8	87.8
How often do you skip your HBP medicine before you go to the doctor?	74.8	15.0	5.3	4.9

**Perceived Barriers to Treatment Compliance Scale (*N*** = **246)**				
**To what extent do you think the following hinder you from complying with your treatment?**	**Not at all**	**To some extent**	**To a larger extent**	**Very much extent**
Ineffectiveness of the medicine to stabilize my blood pressure	63.8	18.3	6.1	11.8
Lack of motivation because I know I cannot be cured even after taking my medications all the time	60.2	13.8	4.1	22.0
Not having enough time to exercise	83.3	12.2	3.7	0.8
Lack of discipline to comply with the dietary restrictions	87.4	9.3	2.4	0.8
Lack of motivation to stop smoking	98.4	0.4	0.8	0.4
Easy access to alcoholic beverages	79.3	16.3	2.4	1.2
Limited financial resources	89.8	7.7	1.6	0.8
Limited access to medications and prescribed foods	93.5	5.7	0.8	0
Side effects	81.3	9.3	7.3	2.1

*Note*: All values are presented as percentages.

Abbreviation: HBP, high blood pressure.

The majority (91.5%) of the respondents were adherent to current medications, whereas 8.5% showed some level of nonadherence to current medication taking

Table 4 shows the correlation of the Perceived Barriers to Medication Adherence Scales and Hill‐Bone Medication Adherence subscale. Perceived noneffectiveness of medication significantly correlated negatively with medication adherence score (*ρ* = −0.50, *p* < 0.001). Also, barriers to alcohol and smoking cessation significantly negatively correlated with medication adherence score (*ρ* = −0.25, *p* = 0.001).

**Table 4:** Correlation between Perceived Barriers to Medication Adherence and Hill‐Bone Medication Adherence subscale

	Medication adherence score			
Perceived barriers	*ρ*	*R* ^2^ score	*F* change	*p* value
Perceived noneffectiveness of medication	−0.50	0.363	138.93	<0.001*
Barriers to medication access	−0.20	0.006	2.12	0.147
Barriers to lifestyle and dietary practices	−0.12	0.010	3.78	0.053
Barriers to alcohol and smoking cessation	−0.25	0.029	11.82	0.001*

*Note*: *ρ* = Spearman's rank correlation coefficient and *R* ^2^ score = coefficient of variations. All *p* values with * are statistically significant.

Cross‐tabulation and Fischer's Exact test are shown in Tables S3 and S4. There was no significant association observed between sociodemographic characteristics and medication nonadherence among the study participants (Table S3). In Table S4, total prescribed medication (*p* = 0.011) and appointment schedules (*p* = 0.031) were significantly associated with adherence status.

Perceived noneffectiveness of medication (odds ratio [OR] = 1.70, 95% confidence interval [CI]: 1.41–2.05, *p* < 0.001) and barriers to alcohol and smoking cessation (OR = 2.26, 95% CI: 1.42–3.58, *p* = 0.001) were associated with increased odds of antihypertensive medication nonadherence. Also, clients who do not know their total prescription were associated with significantly increased odds of medication nonadherence (OR = 4.97, 95% CI: 1.57–15.78, *p* = 0.006). Moreover, clients who associate signs/symptoms of palpitations (OR = 4.18, 95% CI: 1.22–14.35, *p* = 0.023), poor sleep (OR = 4.08, 95% CI: 1.33–1.32, *p* = 0.014), and decreased sexual desire or ability (OR = 4.60, 95% CI: 1.59–13.31, *p* = 0.005) to antihypertensive medication, were more likely to be nonadherent. These associations were not attenuated in the multivariate model.

In a hierarchy regression analysis, as shown in Table S6, perceived barriers to medication noneffectiveness, lifestyle and dietary changes, alcohol and smoking cessation, and perceived symptoms of palpitations and muscle pain were associated with lower medication adherence scores (Table 5).

**Table 5:** Predictors of adherence among the study participants

	*N* (%)	Univariate regression model		Multivariate regression model	
		Predictors	*p* value	aOR (95% CI)	*p* value
Perceived barriers					
Noneffectiveness of medication	N/a	1.70 (1.41–2.05)	<0.001*	1.76 (1.34–2.31)	<0.001*
Barriers to medication access	N/a	1.40 (0.83–2.36)	0.209	1.63 (0.72–3.71)	0.241
Barriers to lifestyle and dietary changes	N/a	1.06 (0.65–1.72)	0.816	0.61 (0.25–1.47)	0.267
Barriers to alcohol and smoking cessation	N/a	2.26 (1.42–3.58)	0.001*	2.83 (1.31–6.13)	0.008*
Sex (male)	10 (47.6)	2.24 (0.91–5.52)	0.081	0.48 (0.14–1.71)	0.259
Perceived side effects of medication					
Tiredness	3 (14.3)	1.40 (0.38–5.08)	0.613	1.53 (0.35–6.82)	0.606
Palpitations	4 (19.0)	4.18 (1.22–14.35)	0.023*	5.82 (1.31–25.80)	0.021*
Muscle pain	4 (19.0)	1.06 (0.34–3.30)	0.925	0.822 (0.22–3.07)	0.770
Headaches	7 (33.3)	1.44 (0.55–3.75)	0.455	0.66 (0.20–2.18)	0.497
Poor sleeping	5 (23.8)	4.08 (1.33–1.32)	0.014*	3.92 (1.09–14.12)	0.036*
Frequent urination	4 (19.0)	3.29 (0.98–11.03)	0.053	1.64 (0.34–7.87)	0.539
Decreased sexual desire or ability	6 (28.6)	4.60 (1.59–13.31)	0.005*	4.74 (0.96–23.28)	0.055
Total prescribed medication					
Don't know	9 (42.9)	4.97 (1.57–15.78)	0.006*	8.81 (2.28–34.0)	0.002*
Yes (<3 medicines)	7 (33.3)	1.79 (0.55–5.85)	0.334	2.10 (0.57–7.74)	0.262
Yes (≥3 medicines)	5 (23.8)	1 (reference)		1 (reference)	

*Note*: All *p* value with * is statistically significant.

Abbreviations: CI, confidence interval; N/a, not applicable; aOR, adjusted odds ratio.

## 4 DISCUSSION

Medication nonadherence presents a significant limitation in combating public health challenges in both developed and developing countries.^13^ It is an active decision of a patient, relatively to misunderstandings of the condition and general disapproval of medication, but mostly taken to facilitate daily life or minimize complications.^14^ On the contrary, adherence to antihypertensive medication is associated with significantly lower total healthcare cost, overall odds of reduced cardiovascular‐related hospitalizations, and lower emergency department visits.^15^ This study assessed the correlates of medication adherence among hypertensive clients in a Ghanaian population. We observed that a significant percentage (approximately 70%) of the hypertensive clients have stopped taking their medication at least within the last 3 months. This action taken by clients has been associated with adverse effects on the medication‐taking behavior of clients. However, medication nonadherence was relatively lower (8.5%) among them. This finding can be attributed to the fact that most (74.9%) of the study participants decidedly skip their BP medicine before their scheduled appointment with the doctor. Moreover, most of the study participants indicated that sometimes but not always, they either forget to take their medications or decide not to take their medication.

Inconsistent with our present findings, some studies have consistently reported a high rate of medication nonadherence among hypertensive clients.^3^, ^5^, ^8^, ^16^ However, Inkster et al.^17^ found that approximately 85% of their participants adhere to their medication regime. This finding suggests that adherence to antihypertensive medication is higher than previously described in some Ghanaian populations.^3^, ^5^ This inconsistency could result from the type of adherence scale used and the defined cut‐off range for defining nonadherence. For most of these scales used in various related studies,^3^, ^5^, ^8^, ^16^ the rationale of the ranking was determined by clinical outcomes and the researcher's expertise. Thus, contributing to a wide range of reported inconsistencies. The advantage of this study is that the 8‐Items Hill‐Bone Medication Adherence Subscale validated for use by this study is one of the most frequently used instruments for measuring high BP medication adherence.^11^, ^18^ Besides, it is the best suitable scale for studies specific to HPT in a predominantly black population.^13^, ^19^, ^20^

The common theme identified as a consistent factor predictive of medication nonadherence was the perceived noneffectiveness of antihypertensive medications. In a multivariate predictive model, perceived noneffectiveness of medication and symptoms of palpitations, poor sleep, and decreased sexual desire or ability associated with medicines were significant influencing factors of medication nonadherence. Grassi et al.^21^ have indicated that greater than half of antihypertensive medication discontinuation can be attributed to adverse effects. We observed that about 48% of the hypertensive clients reported on one or more perceived symptoms associated with the medication. The most symptoms reported by participants include headaches, muscle pain, tiredness, decreased sexual desire or ability, poor sleeping, frequent urination, and palpitations. In a systematic review of qualitative studies by Marshall et al.,^22^ tiredness, urinary frequency, ankle swelling, lethargy, and impotence were mentioned as the most frequent perceived symptoms of hypertensive clients.

These reported signs were associated with medication nonadherence. Gebreyohannes et al.^4^ reported that perceived symptoms of tiredness, muscle pain, and poor sleep attributed to medicines are associated with poor adherence rates. Also, Tedla and Bautista^23^ reported that 85% of hypertensive clients experienced side effects, which was significantly associated with medication nonadherence. Moreover in a systematic review and meta‐analysis by van der Laan et al.^24^ and AlGhurair et al.,^25^ drug side effects were identified as one of the key factors with consistent significant relationships with medication nonadherence. Hypertensive patients frequently report symptoms that are also reported by normotensive patients. Although to a larger extent, these perceived symptoms are true for antihypertensive medication treatment.^26^ A previous study has also stated that increased specific concerns about medication can predict poor adherence, which is similar to our findings.^27^ This finding can be interpreted that, in the Ghanaian society if there is a minor ailment, a patient will just attribute it to their medications without the necessity of reporting to the healthcare provider. These perceptions can create the belief that they are not worried about taking prescribed medicine leading to poor adherence.

The study also observed that patient sociodemographic factors were not associated with medication adherence. In the reports of Inkster et al.,^17^ no significant association was reported between adherence to medication and sociodemographic variables, comorbidities, or the number of antihypertensive drugs taken, which is partly consistent and partly inconsistent with our findings. We also observed that barriers to smoking and alcohol cessation and not knowing total prescribed medications were associated with medication nonadherence. The total number of medications taken by the patient was not associated with adherence. Some studies have partly evaluated these findings.^17^, ^24^ The findings of barriers to smoking and alcohol cessation associated with medication nonadherence have not been justified. In previous unrelated clinical evaluations and computer‐based simulations, alcohol, and smoking affect medication adherence in both HIV‐infected and ‐noninfected clients.^28^, ^29^ This finding in HPT may have clinical implications concerning optimal treatment for hypertensive clients who also smoke or consume alcohol. Thus, better knowledge about HPT and its management and clients' perceived benefits of medication could contribute to better adherence.

The study has a limitation with a small sample size which had an effect on the statistical power of the regression model performed. Also, the Hill‐Bone Scale for measuring adherence to medication was self‐reporting, which may not provide a true picture of actual adherence. Thus, the percentage of the nonadherence rate may either be underestimated or overestimated due to recall and social desirability bias from the clients. This may be on the part of the client trying not to disappoint their doctors or the researchers. Moreover, the cut‐offs used for defining nonadherence both with Hill‐Bone subscale score were predetermined (as an estimate of <80% of the total score). However, there is no standard cut‐off for these measures.

## 5 CONCLUSION

In conclusion, the study observed high adherence to antihypertensive medication among hypertensive clients in a Ghanaian population. We also identified that medicine‐related factors negatively impact adherence to medications. Most importantly, palpitations, poor sleep, and decreased sexual desire or ability significantly predicted lower adherence. Thus, these symptoms could serve as markers to screen outpatients at high risk of nonadherence. Moreover, clients with significant barriers to smoking and alcohol and smoking cessation were likely nonadherent to medications.

## AUTHOR CONTRIBUTIONS

**Yaa Obirikorang**: conceptualization; data curation; formal analysis; funding acquisition; investigation; methodology; project administration; resources; validation; visualization; writing–original draft; writing–review and editing. **Eric Woode**: conceptualization; data curation; formal analysis; methodology; project administration; resources; software; supervision; validation; visualization; writing–review and editing. **Eric Boakye‐Gyasi**: conceptualization; data curation; methodology; supervision; writing–review and editing. **Evans A. Adu**: data curation; formal analysis; methodology; software; validation; writing–original draft; writing–review and editing. **Christian Obirikorang**: conceptualization; data curation; formal analysis; funding acquisition; methodology; resources; validation; writing–review and editing. **Emmanuel Acheampong**: formal analysis; validation; writing–review and editing. **Enoch O. Anto**: formal analysis; validation; writing–review and editing. All authors have read and approved the final version of the manuscript. Dr. Yaa Obirikorang had full access to all of the data in this study and takes complete responsibility for the integrity of the data and the accuracy of the data analysis.

## CONFLICTS OF INTEREST

All authors declare no conflicts of interest.

## Supporting information

Supporting information.

## Data Availability

The authors confirm that the data supporting the findings of this study have all been summarized and are available within the article and/or its supplementary materials.
