# Supplementary material for: Predictors of medication nonadherence among hypertensive clients in a Ghanaian population: Application of the Hill‐Bone and Perceived Barriers to Treatment Compliance Scale
Source: Health Sci Rep. 2022 Apr 13;5(3):e584. doi: 10.1002/hsr2.584 (PMC9059218; doi:10.1002/hsr2.584)
Supplement: Supplementary file 1 — Supporting information. [file HSR2-5-e584-s001.docx]

Supporting data

##### Table S1: Factor solution of the Hill-Bone Compliance to High Blood Pressure Therapy Scale observed in this study

| **Items on the Hill-Bone Scale (14)** | **Unrotated** | | | | **Rotated** | | | |
| --- | --- | --- | --- | --- | --- | --- | --- | --- |
|  | **Factor 1** | **Factor 2** | **Factor 3** | **Factor 4** | **Factor 1** | **Factor 2** | **Factor 3** | **Factor 4** |
| Item 1 | **0.556** | -0.108 | -0.017 | -0.059 | **0.559** | -0.042 | 0.052 | 0.087 |
| Item 2 | **0.569** | -0.287 | 0.256 | 0.467 | **0.464** | 0.024 | -0.056 | **0.686** |
| Item 3 | 0.095 | **0.567** | 0.469 | 0.145 | 0.012 | **0.74** | -0.135 | 0.069 |
| Item 4 | 0.07 | **0.626** | 0.18 | 0.22 | -0.05 | **0.671** | 0.154 | 0.036 |
| Item 5 | 0.48 | 0.253 | 0.176 | 0.037 | **0.430** | 0.365 | 0.034 | 0.082 |
| Item 6 | 0.105 | **0.656** | 0.199 | -0.064 | 0.056 | **0.659** | 0.054 | -0.214 |
| Item 7 | **0.716** | -0.057 | -0.05 | -0.382 | **0.790** | -0.051 | 0.013 | -0.193 |
| Item 8 | **0.753** | -0.084 | 0.143 | -0.142 | **0.774** | 0.061 | -0.07 | 0.085 |
| Item 9 | **0.812** | -0.053 | 0.045 | -0.164 | **0.828** | 0.047 | 0.026 | 0.04 |
| Item 10 | 0.133 | -0.351 | 0.174 | **0.785** | -0.034 | -0.065 | 0.027 | **0.884** |
| Item 11 | **0.739** | -0.104 | -0.027 | 0.01 | **0.716** | -0.007 | 0.118 | 0.179 |
| Item 12 | 0.252 | 0.496 | **-0.578** | 0.272 | 0.093 | 0.248 | **0.804** | -0.034 |
| Item 13 | 0.218 | 0.153 | **-0.753** | 0.254 | 0.093 | -0.135 | **0.822** | 0.018 |
| Item 14 | **0.680** | 0.07 | -0.147 | 0.009 | **0.635** | 0.081 | 0.271 | 0.075 |

***^a^ Correlations between variables and factors > 0.40 are bold; variables are sorted by subscales ‘‘medication taking,’’ ‘‘reduced sodium intake,’’ ‘‘appointment keeping’’ and “a fourth unlabelled scale”.***

##### Table S2: Resolved Items used in this Study

| Variable | **Hill-Bone Subscale** | **Perceived Barriers to Medication Adherence** | | | |
| --- | --- | --- | --- | --- | --- |
|  |  | Perceived medication  benefits | Barriers to accessibility | Barriers to lifestyle and dietary practices | Barriers to alcohol and smoking cessation |
| Number of Items | 8 | 3 | 2 | 2 | 2 |
| Cronbach’s Alpha | 0.701 | 0.800 | 0.601 | 0.632 | 0.621 |
| Inter-item correlation | 0.243 | 0.701 | 0.432 | 0.534 | 0.492 |
| Possible raw score range | 8-32 | 3-12 | 2-8 | 2-8 | 2-8 |
| Observed raw score | 19-32 | 3-12 | 2-7 | 2-7 | 2-10 |
| Mean | 29.0 | 4.8 | 2.2 | 2.4 | 2.3 |

##### Table S3: Association of Socio-demographic Variables with Medication Adherence

| **Variable** | **Medication adherence scale** | | **Fishers Exact Test** |
| --- | --- | --- | --- |
|  | **Non-adherence**  **(N=21)** | **Adherence**  **(N=225)** |  |
| **Age (years)** |  |  | 0.220 |
| <50 | 2 (9.5) | 40 (17.8) |  |
| 50-59 | 12 (57.1) | 79 (35.1) |  |
| 60-69 | 6 (28.6) | 90 (40.0) |  |
| 70-79 | 1 (4.8) | 16 (7.1) |  |
| **Gender** |  |  | 0.086 |
| Female | 11 (52.4) | 160 (71.1) |  |
| Male | 10 (47.6) | 65 (28.9) |  |
| **Marital Status** |  |  | 0.134 |
| Single | 5 (23.8) | 21 (9.3) |  |
| Married | 14 (66.7) | 174 (77.3) |  |
| Widowed | 2 (9.5) | 30 (13.3) |  |
| **Level of education** |  |  | 0.199 |
| No formal education | 4 (19.00 | 37 (16.4) |  |
| Basic School | 13 (61.9) | 99 (44.0) |  |
| High school | 3 (14.1) | 33 (14.7) |  |
| Tertiary | 1 (4.8) | 56 (24.9) |  |
| **Occupation** |  |  | 0.150 |
| Government employee | 1 (4.8) | 6 (20.4) |  |
| Retired | 2 (9.5) | 41 (18.2) |  |
| Self-employed | 15 (71.4) | 116 (51.6) |  |
| Unemployed | 3 (14.3) | 22 (9.8) |  |
| **Religion** |  |  | 1.00 |
| Christian | 19 (90.5) | 200 (88.9) |  |
| Muslim | 2 (9.5) | 25 (11.1) |  |
| **Duration on treatment** |  |  | 0.779 |
| < 1 year | 0 | 14 (6.2) |  |
| 1-3 years | 2 (9.5) | 19 (8.4) |  |
| 4-7 years | 2 (9.5) | 36 (16.0) |  |
| 8-10 years | 8 (38.1) | 72 (32.0) |  |
| >10 years | 9 (42.9) | 84 (37.3) |  |

##### Table S4: Association of Medication History with Medication Adherence

| **Variable** | **Medication Adherence Scale** | | **Fishers Exact Test** |
| --- | --- | --- | --- |
|  | **Non-adherence**  **(N= 21)** | **Adherence**  **(N=225)** |  |
| **Knowledge of current medication** |  |  | 0.112 |
| No | 15 (71.4) | 118 (52.4) |  |
| Yes | 6 (28.6) | 107 (47.6) |  |
| **Total prescribed medication** |  |  | **0.012** |
| Don’t know | 9 (42.9) | 38 (16.9) |  |
| <3 medicines | 7 (33.3) | 82 (36.4) |  |
| 3 or more medicines | 5 (23.8) | 105 (46.7) |  |
| **Prescription pattern** |  |  | 0.341 |
| Monotherapy | 1 (4.8) | 9 (4.0) |  |
| Fixed dose combination | 3 (14.3) | 31 (13.8) |  |
| Two combinations | 8 (38.1) | 81 (36.0) |  |
| Three combinations | 2 (9.5) | 66 (29.3) |  |
| Four combinations | 5 (23.8) | 26 (11.6) |  |
| Five combinations | 2 (9.5) | 12 (5.3) |  |
| **Appointment periods** |  |  | **0.024** |
| <Every 3 months | 5 (23.8) | 17 (7.6) |  |
| Every 3 | 11 (52.4) | 119 (52.9) |  |
| Every 4 months and beyond | 5 (23.8) | 89 (39.6) |  |
| **Perceived signs/symptoms of medication** |  |  | **0.624** |
| No | 7 (33.3) | 120 (53.3) |  |
| Yes | 14 (66.7) | 105 (46.7) |  |

| **Table S5: Model Summary of Hierarchical regression analysis of factors predictive of medication non-adherence** | | | | | | | | | |  |  |
| --- | --- | --- | --- | --- | --- | --- | --- | --- | --- | --- | --- |
| Model | R | R Square | Adjusted R Square | Std. Error of the Estimate | Change Statistics | | | | | | |
|  |  |  |  |  | R Square Change | F Change | df1 | df2 | Sig. F Change | |  |
| 1 | .638^a^ | .407 | .397 | 1.74864 | .407 | 41.369 | 4 | 241 | **.000** | |  |
| 2 | .640^b^ | .410 | .397 | 1.74847 | .003 | 1.046 | 1 | 240 | .307 | |  |
| 3 | .670^c^ | .449 | .421 | 1.71445 | .039 | 2.374 | 7 | 233 | **.023** | |  |
| 4 | .680^d^ | .462 | .432 | 1.69704 | .013 | 5.805 | 1 | 232 | **.017** | |  |

1. Predictors: (Constant), Smoking_Alcohol, Acessibility, Diet_Excercise, Medication

2. Predictors: (Constant), Smoking_Alcohol, Acessibility, Diet_Excercise, Medication, sex

3. Predictors: (Constant), Smoking_Alcohol, Acessibility, Diet_Excercise, Medication, sex, sleep poorly, head, palp, mpain, frq urine, tired, libido

4. Predictors: (Constant), Smoking_Alcohol, Acessibility, Diet_Excercise, Medication, sex, sleep poorly, head, palp, mpain, frq urine, tired, libido, current medication

**Table S6: Coefficients of the final hierarchical regression model predictive of medication non-adherence**

| Model | Unstandardized Coefficients | | Standardized Coefficients | t | Sig. |
| --- | --- | --- | --- | --- | --- |
|  | Betta | Std. Error | Beta |  |  |
| **(Constant)** | **32.143** | **0.627** |  | **51.273** | **0.000** |
| non-effectiveness of medication | -0.502 | 0.049 | -0.57 | -10.155 | **0.000** |
| Barriers to medication access | -0.269 | 0.178 | -0.077 | -1.514 | 0.131 |
| Barriers to lifestyle and dietary changes | 0.264 | 0.131 | 0.102 | 2.011 | **0.045** |
| Barriers to alcohol and smoking cessation | -0.488 | 0.13 | -0.197 | -3.743 | **0.000** |
| Sex (Male) | 0.051 | 0.283 | 0.01 | 0.18 | 0.857 |
| Tiredness | 0.025 | 0.37 | 0.004 | 0.068 | 0.945 |
| Palpitations | -1.066 | 0.454 | -0.117 | -2.345 | **0.020** |
| Muscle pain | -0.663 | 0.304 | -0.114 | -2.183 | **0.030** |
| Headaches | 0.175 | 0.259 | 0.034 | 0.677 | 0.499 |
| Poor sleeping | -0.405 | 0.397 | -0.05 | -1.022 | 0.308 |
| Frequent urination | 0.468 | 0.445 | 0.056 | 1.05 | 0.295 |
| Decreased sexual desire or ability | -0.038 | 0.453 | -0.005 | -0.084 | 0.933 |
| current medication | 0.352 | 0.146 | 0.119 | 2.409 | **0.017** |
